# Specifics of professional deformations of personality in military personnel

**DOI:** 10.1192/j.eurpsy.2021.1190

**Published:** 2021-08-13

**Authors:** D. Boyarinov, Y. Novikova, L. Gubaidulina, A. Kachina, V. Barabanshchikova

**Affiliations:** Faculty Of Psychology, Lomonosov Moscow State University, Moscow, Russian Federation

**Keywords:** Professional Deformations, Military Personnel, Chronic Stress, Burnout Syndrome

## Abstract

**Introduction:**

Work experience develops not only professional skills, but also affects people’s personal characteristics. A long period of intensive work usually promotes the development of professional deformations. Prolonged exposure in stressful conditions is a risk factor for developing professional deformations. Military service is intense and stressful. Armed forces personnel work is related to unquestioning execution of orders and extreme working conditions (Kozlova, 2013). These risk factors may cause professional deformations of personality. The study was supported by the RFBR #19-013-00799 А.

**Objectives:**

Influence of working activity on professional deformations development in military personnel.

**Methods:**

The research involved 708 participants, the sample consisted only of men. Average age 20.3 years (min – 18, max – 32). They fulfilled 2 standardized questionnaires: Managerial stress survey — MSS (Leonova, 2007), The Sixteen Personality Factor Questionnaire (ed. Kapustina, 2001).

**Results:**

Based on the findings the following outcome can be seen: the high scores of the Acute stress (M=45,7; SD=5,2), medium level of Chronic stress (M=43,6; SD=6,5), and Professional deformations (M=43,4; SD=7,1). In particular it turned out that burnout syndrome (M=46,1; SD=6,9), neurotic reactions (M=45,4; SD=7,0) and behavioral risk factors (M=46,7; SD=8,7) are high.

**Conclusions:**

In that way, we can assume that Professional deformations aren’t developed by military personnel in our research. That point of view confirms on Acute and Chronic stress level. Despite the fact that there are some behavioral risk factors. That can cause a reduction of work efficiency and a decline in the health level of military personnel.

